# Distance Walked and Run as Improved Metrics over Time-Based Energy Estimation in Epidemiological Studies and Prevention; Evidence from Medication Use

**DOI:** 10.1371/journal.pone.0041906

**Published:** 2012-08-20

**Authors:** Paul T. Williams

**Affiliations:** Lawrence Berkeley National Laboratory, Life Sciences Division, Berkeley, California, United States of America; University of Bath, United Kingdom

## Abstract

**Purpose:**

The guideline physical activity levels are prescribed in terms of time, frequency, and intensity (e.g., 30 minutes brisk walking, five days a week or its energy equivalence) and assume that different activities may be combined to meet targeted goals (exchangeability premise). Habitual runners and walkers may quantify exercise in terms of distance (km/day), and for them, the relationship between activity dose and health benefits may be better assessed in terms of distance rather than time. Analyses were therefore performed to test: 1) whether time-based or distance-based estimates of energy expenditure provide the best metric for relating running and walking to hypertensive, high cholesterol, and diabetes medication use (conditions known to be diminished by exercise), and 2) the exchangeability premise.

**Methods:**

Logistic regression analyses of medication use (dependent variable) vs. metabolic equivalent hours per day (METhr/d) of running, walking and other exercise (independent variables) using cross-sectional data from the National Runners' (17,201 male, 16,173 female) and Walkers' Health Studies (3,434 male, 12,384 female).

**Results:**

Estimated METhr/d of running and walking activity were 38% and 31% greater, respectively, when calculated from self-reported time than distance in men, and 43% and 37% greater in women, respectively. Percent reductions in the odds for hypertension and high cholesterol medication use per METhr/d run or per METhr/d walked were ≥2-fold greater when estimated from reported distance (km/wk) than from time (hr/wk). The per METhr/d odds reduction was significantly greater for the distance- than the time-based estimate for hypertension (runners: P<10^−5^ for males and P = 0.003 for females; walkers: P = 0.03 for males and P<10^−4^ for females), high cholesterol medication use in runners (P<10^−4^ for males and P = 0.02 for females) and male walkers (P = 0.01 for males and P = 0.08 for females) and for diabetes medication use in male runners (P<10^−3^).

**Conclusions:**

Although causality between greater exercise and lower prevalence of hypertension, high cholesterol and diabetes cannot be inferred from these cross-sectional data, the results do suggest that distance-based estimates of METhr/d run or walked provide superior metrics for epidemiological analyses to their traditional time-based estimates.

## Introduction

Physical activity recommendations are defined in terms of duration (time), frequency, and intensity (e.g., i.e., 150 minutes per week of moderate aerobic physical activity or 75 minutes of vigorous aerobic physical activity per week), and they assume that different activities can be combined to meet targeted goals (exchangeability premise) [1]–[6]. The recommendations are also specified in terms of energy expenditure, which is calculated as the product of the time spent performing each activity and its intensity as represented by metabolic equivalents (METs, representing their X-fold increase in energy expenditure relative to sitting at rest, 1 MET = 3.5 ml O_2_•kg^−1^•min^−1^ [1]). The energies expended at each activity are then summed and compared to target levels (e.g., 450 to 750 METminutes per week for health benefits [3]).

Energy expended by walking and running may be calculated from: 1) time and intensity (i.e., pace or minutes per mile) as in as in the physical activity recommendations and in most epidemiologic studies [7], or 2) distance and intensity as reported in the National Runners' and Walkers' Health Studies [8]–[23]. A number of health benefits have been ascribed to longer weekly distances run (equivalent to running energy expenditure [24]) or walked, benefits that continue to accrue at higher activity levels and for additional disease endpoints than reported for time-based estimates of exercise energy expenditure [8]–[23]. In part, this might be due to self-reported distance in habitual runners and walkers being more reliably reported than self-reported intensity and duration in other populations. For example, it has been shown that that BMI and regional adiposity were more strongly related the distance-based calculations of energy expended by running and walking than their corresponding time-based calculation [24], [25]. However, others have reported that obesity per se may bias self-reported physical activity [26]. Extending these finding to disease conditions not known to affect exercise participation such as hypertension and high-cholesterol, would strengthen the results. Moreover, hypertension, hypercholesterolemia, and diabetes are major risk factors for coronary heart disease [27], and coronary heart disease risk reduction is a major benefit of both running and walking [2], [3]. A direct comparison of a time-based and distance-based estimation of energy expenditure in their relationship to hypertension, high-cholesterol and diabetes in runners and walkers has not been previously reported.

Therefore, this report compares energy expenditure from reported distance vs. reported time walked (or run) to hypertensive, high cholesterol, and diabetes medication use in order to determine which calculation of energy expenditure, time-based or distance-based, shows the strongest relationship to disease. The metric showing the greatest effect and the strongest statistical significance may be the metric most closely related to the quality of exercise that confers its health benefit, although causality cannot be inferred from these cross-sectional associations. It may also be the most reliably reported, since the error in measuring the independent variable (e.g., energy expenditure) reduces statistical significance and biases the logistic regression coefficient towards the null hypothesis [28]. The metric showing the most significant relationship to health outcomes might also be the best metric for prescribing exercise to the public.

In addition, this report seeks to test the exchangeability premise, i.e., whether different physical activities can be exchanged to produce the same health benefits so long as total energy expended remains the same. Physical activities differ in their intensities, with those expending ≥6 METs being classified as vigorously intense (e.g., running), and those expending between 3 and 6 METs being classified as moderately intense (e.g., walking) [1]. It has been shown previously that BMI and regional adiposity were more strongly related to energy expended by running than by moderate or other vigorous exercise [24]. However, it is not known whether the purported obesity-related bias affecting self-reported physical activity [26] might affect these analyses as well. Therefore, this report also tests whether the odds for hypertensive, high cholesterol, and diabetes medication use are the same for equivalent energy spent: 1) running vs. walking, 2) running or walking vs. all other exercise, 3) running vs. all other vigorous exercise, and 4) walking vs. all other moderate exercise.

## Methods

The sample used in these cross-sectional analyses was obtained from a partial re-survey of the National Runners' and Walkers' Health Studies in 2006 [24], [25]. The goal of the resurvey was to provide a base population of approximately 50,000 runners and walkers for a proposed clinical trial, rather than obtaining a high response rate for a more manageable sample size. The runners and walkers completed a four page survey on running history (average weekly mileage over the preceding 5 years, minutes required to run a mile, frequency of runs per week >10 min, longest usual run), height, weight and body circumferences, diet (vegetarianism and the current weekly intakes of alcohol, red meat, fish, fruit), current and past cigarette use, and history of diseases. Running and walking distances during the current year were reported in miles per week, which was converted to kilometers per day. Previously, we have reported strong correlations between repeated questionnaires for self-reported running distances (r = 0.89) [29]. In addition, the questionnaires asked “On average, how many hours per week do you spend running ___, walking ____, swimming ____, cycling ____, other exercise (describe in detail) ____.” and “During your usual run (walk), how many minutes does it take to run (walk) one mile?”

The reported exercise intensities were classified as light- (<3 METs), moderate- (3 to 6 METs) and vigorously intense (>6 METs) [1], [3]. Time-based energy expenditures (METhr/d) of total, vigorous, moderate, and light intensity exercise were calculated as the product of the average number hours per day spent on each exercise and the estimated energy expenditure for the exercise as listed in the 2000 compendium of physical activities [7]. The compendium gives the MET expenditures for running that translate into an exercise dose that is solely a function of distance (Running_Distance_ = 1.02 MET hour per km [24]). Time-based calculation of METhr/d run (Running_Time_) was computed as the product of the MET values published in the compendium and the average hours run per day.

For walking, the compendium gives the MET-values for walking on a firm flat surface as 2.5 METs at 2.0 mph (slow pace), 3.0 METs at 2.5 mph, 3.3 METs at 3.0 mph (moderate pace), 3.8 METs at 3.5 mph (brisk pace), 5 METs at 4 mph (very brisk pace), and 6.3 METs at 4.5 mph (very, very brisk pace). We assigned walking an energy expenditure of 3.8 METs for any walkers who did not provide their usual walking pace (i.e., the compendium value for walking for exercise, 3.5 mph [7]). The time-based calculation of METhr/d walked was computed as the product of the average hours walked per day and the MET value corresponding to their reported pace (Walking_Time_). The distance-based calculation of METhr/d walked was computed by converting the reported distance into duration (i.e., distance/mph) and then calculating the product of the average hours walked per day and the MET value corresponding to their reported pace (Walking_Distance_). Reported activities that omitted duration were assigned the mean duration for that activity. The protocol for this study was reviewed and approved by the University of California Berkeley committee for the protection of human subjects, and all subjects provided a signed a statement of informed consent.

### Statistical analyses

Statistical analyses were performed using Stata (StataCorp LP, College Station TX, version 11). Table 1 presents means±SD for all variables assessed; all other statistics are expressed as mean±SE or coefficients±SE. Logistic regression analyses were used to estimate the relationships of medication use to METhr/d of running, walking, and other exercise. Covariates included adjustments for age (age and age^2^), education, current smoking status, and intakes of meat, fruit, and alcohol. As these data are observational and cross-sectional, they cannot prove causality. The use of the terminology “increasing METhr/d” in reference to the independent variable and “decreasing medication use” in reference to the dependent variables pertain only to their mathematical functional relationship and are not intended to imply change over time.

**Table 1:** Sample characteristics (±SD).

	Runners		Walkers	
	Male	Female	Male	Female
Sample (N)	17201	16173	3434	12384
Age (years)	54.56±11.04	47.46±10.69	66.97±11.26	58.79±12.09
Education (years)	16.79±2.49	16.36±2.31	16.27±2.78	15.29±2.55
Smokers (%)	1.15	1.50	2.85	2.93
Meat (servings/day)	0.46±0.45	0.31±0.44	0.47±0.48	0.39±0.41
Fruit (pieces/day)	1.52±2.03	1.59±1.52	1.59±2.19	1.60±1.32
Alcohol (g/day)	11.22±14.96	7.14±9.65	9.13±13.70	5.20±10.56
BMI (kg/m^2^)	24.83±3.17	22.27±3.15	27.00±4.73	26.07±5.61
Medication use (%)				
Hypertension	16.99	7.27	43.10	27.76
High cholesterol	24.69	10.36	43.19	29.97
Diabetes	1.54	0.78	10.75	4.97
Running				
Distance (Km/d)	4.00±3.10	3.48±2.98		
Duration (hours)	0.49±0.46	0.46±0.48		
Running_Distance_ (METhr/d from self-reported distance)	4.02±3.17	3.48±3.04		
% of total METs	54.78±33.26	44.94±32.12		
Running_Time_ (METhr/d from self-reported duration)	5.58±5.39	4.96±5.29		
Walking				
Distance (Km/d)			2.88±2.44	2.67±2.33
Duration (hours)			0.66±0.67	0.63±0.64
Walking_Distance_ (METhr/d from self-reported distance)			2.11±1.92	1.98±1.86
% of total METs			63.86±36.20	60.06±35.81
Walking_Time_ (METhr/d from self-reported duration)			2.78±2.93	2.71±2.91
% of total METs			63.65±36.35	61.48±35.89
Other exercise METhr/d				
Total	3.96±5.01	4.70±4.88	2.49±4.26	2.44±3.74
Other vigorous intensity	2.28±4.31	2.56±4.02	1.61±3.51	1.60±3.24
Light and moderate intensity	1.68±2.49	2.14±2.65		
Other moderate intensity			0.80±2.19	0.74±1.76
Light intensity			0.08±0.71	0.09±0.45

Two different tests were used to assess whether the distance-based calculation of METhr/d run or walked differed from its traditional time-based calculation in predicting medication use. Both use a model that simultaneously includes separate regression terms for METhr/d calculated from time and METhr/d calculated from distance, e.g.: 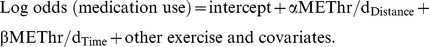
1) The significance of the individual coefficients that test whether adding the distance-based estimate significantly improves the model over one that includes only the time-based estimate (i.e., α = 0), and correspondingly, whether adding the time-based estimate significantly improves the model over one that includes only the distance-based estimate (i.e., β = 0). 2) Direct comparison of the equivalence of the coefficients (i.e., α = β) by linear contrasts.

The exchangeability premise, i.e., whether METhr/d from running (or walking) differs from those of other exercise, was tested as the significance of the contrast α = β, in the model:
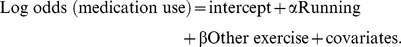
The runners and walkers were also compared directly in their combined sample using the model:
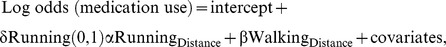
where “Running(0,1)” is an indicator variable that distinguishes overall risk difference in the runners and the walkers, the covariate are assumed to have the same effects in the runners and walkers, and the equivalence of runners vs. walkers is tested by the significance of α = β.

## Results

Recruitment was targeted at obtaining updated survey questionnaires on approximately fifty thousand subjects. These represented approximately a third of the original walker (33.2%), and one-half of the original runner surveyed (51.7%). Compared to non-responders, those that responded were slightly less likely to be male (responders vs. non-responders, runners: 45.1% vs. 51.4%; walkers: 19.7% vs. 21.8%), younger (mean±SE, runners: 40.1±11.4 vs. 45.2±11.7; walkers: 51.3±14.2 vs. 54.1±12.3 years), slightly less educated (runners: 16.0±2.6 vs. 16.6±2.6; walkers: 15.0±2.6 vs. 15.5±2.6 years), weighed slightly more (runners: 23.1±3.3 vs. 23.0±3.0; walkers: 26.0±5.3 vs. 25.5±4.9 kg/m^2^), were less likely to report taking medications for blood pressure, hypertension, or diabetes (runner 5.5 vs. 7.2%, walkers: 20.0 vs. 21.7%), and reported approximately the same number of km/day run if a runner (4.9±3.1 vs. 5.0±3.0 km/d) or walked if a walker (2.9±2.1 vs. 3.0±2.0 km/d) as reported on their original questionnaire.

Table 1 presents the characteristics of the sample used in the analyses. The walkers were generally older and heavier than the runners, and the men older and heavier than the women. The men also consumed more meat and alcohol, and ran somewhat longer distances. Estimated METhr/d of running activity was 38% greater when calculated from self-reported time (hours) and intensity (pace) than from self-reported weekly distance in men, and 43% greater in women. Estimated METhr/d of walking activity was 31% greater when calculated from time and intensity vis-à-vis from distance in men, and 37% greater in women. Medication use for treating hypertension, high cholesterol, and diabetes were substantially greater for walkers than runners, perhaps reflecting the walkers' older age and greater BMI. Walking represented only 33.2% of non-running exercise in male runners, and 36.5% in female runners. Running represented only 11.3% of non-walking exercise in male walkers, and 7.4% in female walkers.

### Running

#### Distance-based METhr/d run vs. other exercise

Consistent with our previously published reports [8], [9], Table 2 shows that the odds for hypertension, high cholesterol, and diabetic medication use all decreased significantly in relation to METhr/d run in both men and women. When estimated from self-reported distance, METhr/d run had a substantially stronger relationship to medication use than METhr/d from other exercise. For example, the odds for hypertensive medication use declined 7.9% (i.e. 100×(1-0.921)) per METhr/d run but only 1.3% per METhr/d from other exercise, a 6-fold difference. In women, the difference was 3.6-fold. Compared to other exercise, the estimated effects per METhr/d run in men and women were 5.7- and 16.8-fold greater for high cholesterol medication use, respectively, and 47.4- and 5.1-fold greater for diabetes medication use. The significant differences in the coefficients for METhr/d of running vs. other exercise varied from P = *2.0×10^−5^* to <10^−15^ in men, and from P = 0.01 to *1.5×10^−10^* in women. The differences were slightly less significant when METhr/d run were compared to other vigorous exercise instead of all other exercise (i.e., all light, moderate and other vigorously intense exercise combined).

**Table 2:** Odds ratio (95% confidence interval) for hypertensive, high cholesterol, and diabetes medication use versus METhours per day of running and other physical activities.

	Dependent variables:		
	Hypertension	High cholesterol	Diabetes
**Males**			
Distance-based estimate			
Running_Distance_	0.92¶	0.93¶	0.89§
	(0.91, 0.93)	(0.92, 0.94)	(0.85, 0.94)
Other exercise	0.99†	0.99†	1.00
	(0.98, 1.00)	(0.98, 1.00)	(0.97, 1.02)
*Running_Distance_ vs. other exercise*	*P<10^−15^*	*P<10^−15^*	*P = 2.0×10^−5^*
Running_Distance_	0.92¶	0.93¶	0.90§
	(0.91, 0.94)	(0.92, 0.94)	(0.86, 0.94)
Other vigorous exercise	0.98‡	0.98‡	0.97
	(0.97, 0.99)	(0.97, 0.99)	(0.94, 1.01)
Light & moderate exercise	1.00	0.99	1.04
	(0.99, 1.02)	(0.98, 1.01)	(1.00, 1.08)
*Running_Distance_ vs. other vigorous*	*P = 2.2×10^−10^*	*P = 9.3×10^−14^*	*P = 0.006*
Time-based estimate			
Running_Time_	0.96§	0.96¶	0.96†
	(0.95, 0.97)	(0.96, 0.97)	(0.94, 0.99)
Other exercise	0.99*	0.99*	1.00
	(0.98, 1.00)	(0.98, 1.00)	(0.98, 1.03)
*Running*_Time_ *vs. other exercise*	*P = 2×10^−5^*	*P = 6.7×10^−7^*	*P = 0.03*
Running_Time_	0.96§	0.96¶	0.97*
	(0.95, 0.97)	(0.96, 0.97)	(0.94, 0.99)
Other vigorous exercise	0.98‡	0.99†	0.98
	(0.97, 0.99)	(0.98, 1.00)	(0.94, 1.01)
Light & moderate exercise	1.01	1.00	1.05*
	(0.99, 1.02)	(0.99, 1.01)	(1.01, 1.09)
*Running*_Time._ *vs. other vigorous*	*P = 0.02*	*P = 0.0003*	*P = 0.63*
**Females**			
Distance-based estimate			
Running_Distance_	0.94§	0.93§	0.88‡
	(0.92, 0.96)	(0.91, 0.95)	(0.81, 0.94)
Other exercise	0.98*	1.00	0.98
	(0.97, 1.00)	(0.98, 1.01)	(0.94, 1.02)
*Running_Distance_ vs. other exercise*	*P = 0.001*	*P = 1.5×10^−10^*	*P = 0.01*
Running_Distance_	0.94§	0.93§	0.88‡
	(0.92, 0.96)	(0.91, 0.95)	(0.82, 0.95)
Other vigorous exercise	0.98†	0.99	0.96
	(0.96, 0.99)	(0.97, 1.00)	(0.91, 1.02)
Light & moderate exercise	0.99	1.01	1.00
	(0.97, 1.02)	(0.99, 1.03)	(0.94, 1.07)
*Running_Distance_ vs. other vigorous*	*P = 0.01*	*P = 6.7×10^−7^*	*P = 0.06*
Time-based estimate			
Running_Time_	0.98‡	0.96§	0.87§
	(0.96, 0.99)	(0.95, 0.97)	(0.82, 0.92)
Other exercise	0.98*	1.00	0.98
	(0.97, 1.00)	(0.99, 1.01)	(0.93, 1.02)
*Running*_Time_ *vs. other exercise*	*P = 0.46*	*P = 1.4×10^−5^*	*P = 0.002*
Running_Time_	0.98‡	0.96§	0.87§
	(0.97, 0.99)	(0.95, 0.97)	(0.83, 0.93)
Other vigorous exercise	0.98†	0.99	0.96
	(0.96, 0.99)	(0.97, 1.00)	(0.91, 1.02)
Light & moderate exercise	1.00	1.02	1.00
	(0.97, 1.02)	(1.00, 1.04)	(0.93, 1.06)
*Running*_Time._ *vs. other vigorous*	*P = 0.98*	*P = 0.009*	*P = 0.02*

Adjusted for age, education, current smoking status, and intakes of meat, fruit, and alcohol.

Significance of the regression coefficients coded.

* P<0.05;

† P<0.01;

‡ P<0.001;

§ P<0.0001;

¶ P<10^−15^,

in the model: Log odds (medication) = intercept+αRunning_Distance_+βOther exercise+covariates, or Log odds (medication) = intercept+αRunning_Time_+βOther exercise+covariates.

Abbreviations: BMI, body mass index; MET, metabolic equivalents of energy expenditure; Running_Distance_, metabolic equivalent hr/d from running as estimated from self-reported distance, Running_Time_, metabolic equivalent hr/d from running as estimated from self-reported duration.

#### Distance vs. time estimates of METhr/d run

Table 2 shows that the percent reductions in the odds for men's medication use per METhr/d run were over twice as great when estimated from reported km/day than when estimated from time and intensity. For example, the 7.9% decline in hypertensive medication use per METhr/d for reported distance run was 2.2-fold greater than its 3.6% reduction per METhr/d run for its traditional estimate based on time and intensity. In women, the corresponding comparison was a 2.7–fold difference. Similarly, for cholesterol medication use, the men's and women's declines per METhr/d run were 2-fold greater when calculated from reported distance than from reported time. The percent odds reduction for diabetes medication use per METhr/d was 3-fold greater when calculated from self-reported distance than self-reported duration in men. In women, however, the declines in the odds for women's diabetes medication use were not different when estimated from distance and when estimated from time.

#### Time-based estimate of METhr/d run vs. other exercise

The preceding analyses of running vs. other exercise were based on a distance-based metric for METhr/d run, and a time-based metric for METhr/d of other exercise. The comparisons are germane to understanding the strengths of the National Runners' Health Study results (distance based) vs. composite estimates of physical activity from representative population samples (time based). With respect to evaluating the exchangeability premise, the METhr/d for running and other exercise must be estimation using a common metric (time). The table shows that when all activities, both running and nonrunning, were estimated from self-reported time and intensity, the associations were significantly stronger (P = 0.03 to 6.7×10^−7^) for running than non-running activities in men, and significantly stronger for hypercholesterolemia (P = 1.4×10^−5^) and diabetes medication use (P = 0.002) in women. In most cases the difference is not simply attributable to running being vigorously intense, and the “other exercise” category including moderate and light intensity exercise. Specifically, for hypertension in both sexes, hypercholesterolemia in men, and hypercholesterolemia and diabetes in women, METhr/d from running was more strongly related to medication use than METhr/d from other vigorous activities, in contradiction to the exchangeability premise. Light and moderate-intensity exercise was not inversely associated with medication usage.

#### Independent effects of time vs. distance estimates of METhr/d run

**Figure 1:**
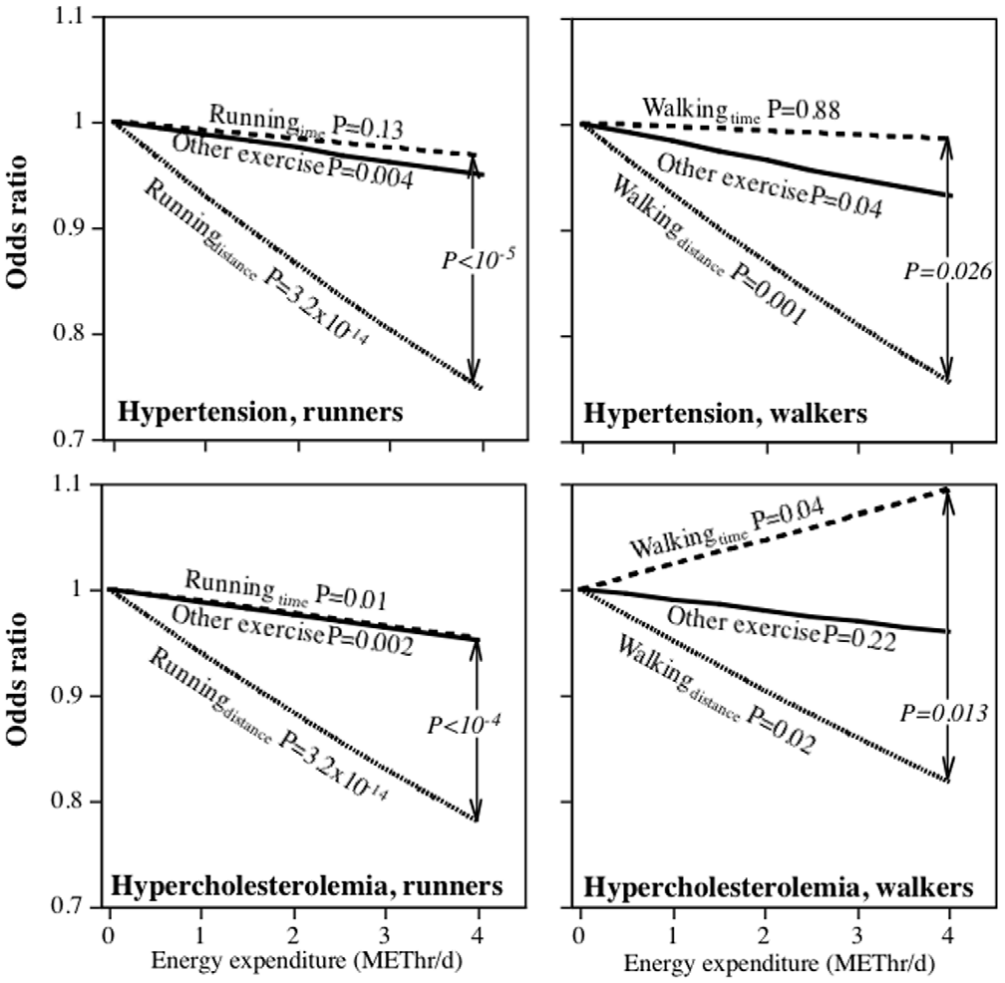
Odds reduction in men's medication use to control for hypertension and high cholesterol per METhr/d energy expenditure by running (National Runners' Health Study) or walking (National Walkers' Health Study). Significance levels presented for α = β, α = γ, and β = γ in the models: log odds(medication use) = intercept+αRunning_Distance_+βRunning_Time_+γOther exercise_Time_+covariates, and log odds(medication use) = intercept+αWalking_Distance_+βWalking_Time_+γOther exercise_Time_+covariates.

**Figure 2:**
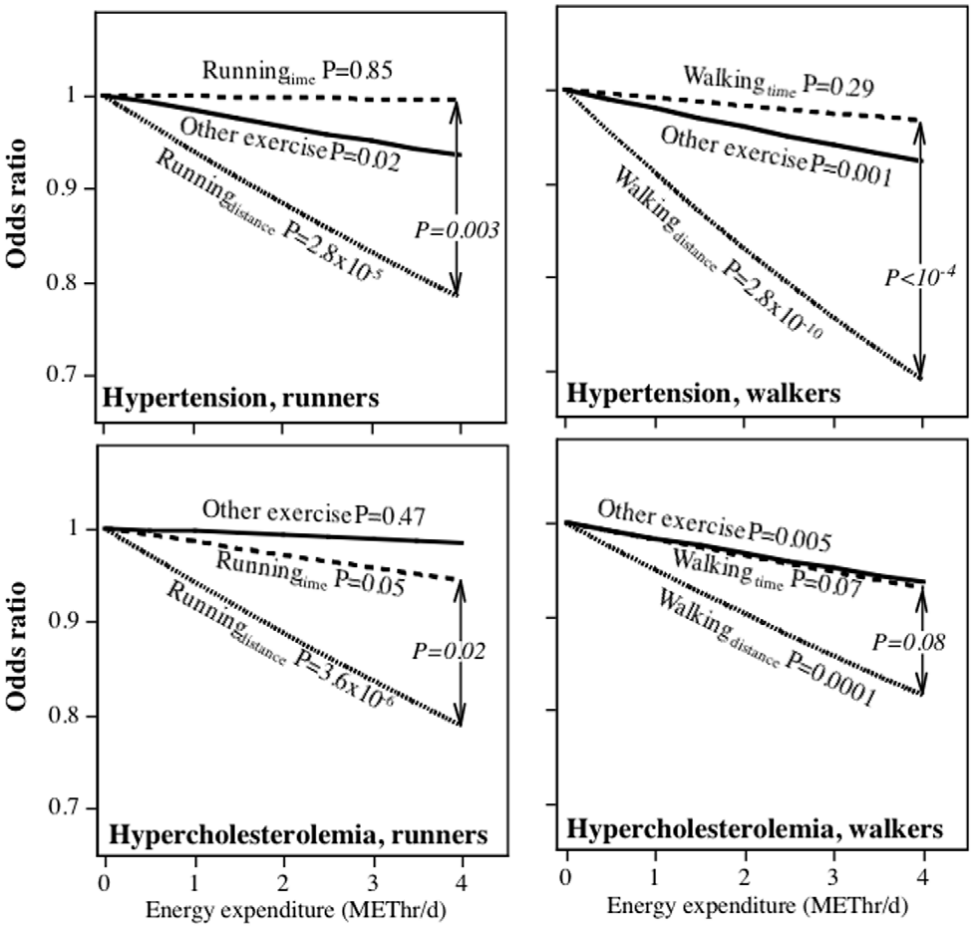
Odds reduction in women's medication use to control for hypertension and high cholesterol per METhr/d energy expenditure by running (National Runners' Health Study) or walking (National Walkers' Health Study). See legend to Figure 1 for model.

**Figure 3:**
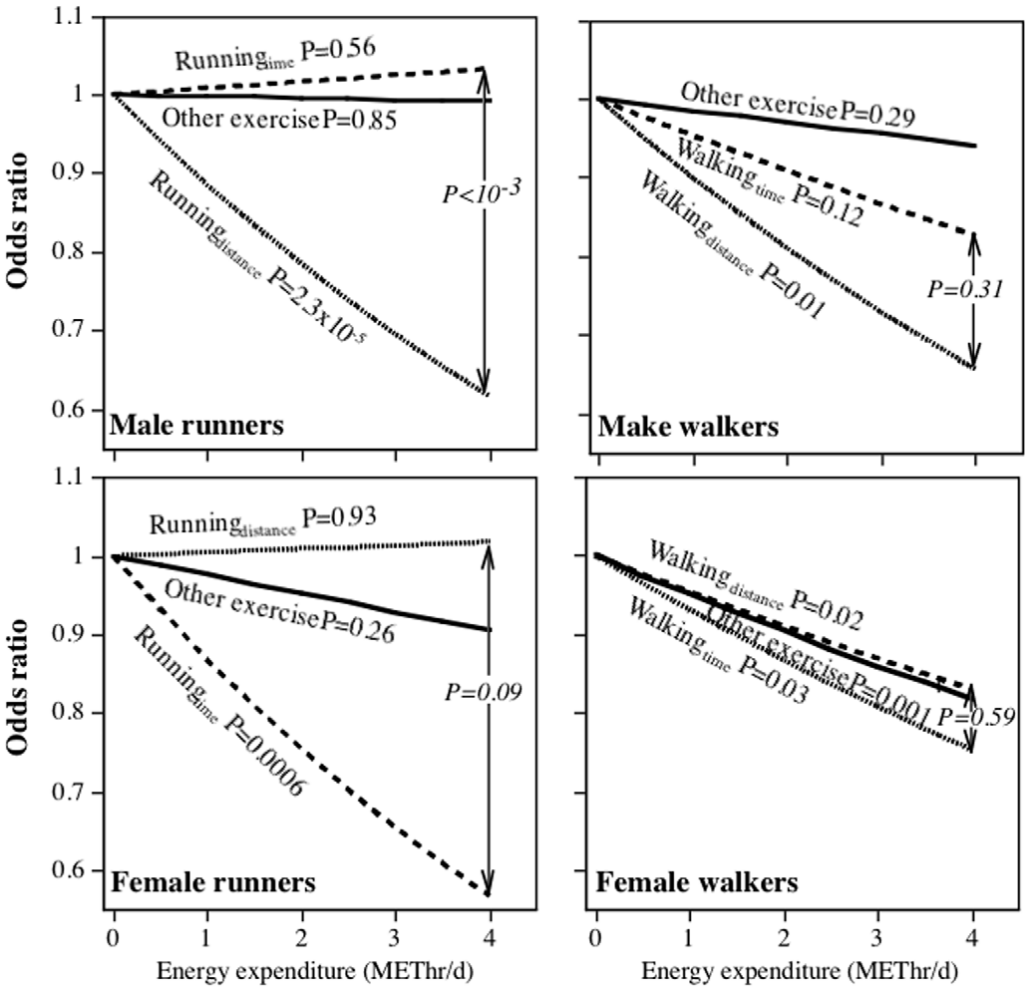
Odds reduction in medication use to control diabetes per METhr/d energy expenditure by running (National Runners' Health Study) or walking (National Walkers' Health Study). See legend to Figure 1 for model.

Table 2 examines the effects of exercise on medication use when METhr/d run is estimated by *either* time or distance. This was done in order to compare running to other (non-running) exercise. Figures 1–3 display the logistic regression analyses for the odds of medication use when the distance- and time-based calculations of METhr/d run were included simultaneously in the model, thereby permitting a direct comparison of their associated odds reduction. In contrast to the tables, the figures present a direct statistical test of whether distance-based or time-based calculations shows the strongest association with medication use. For example, the odds for hypertensive medication use in men decreased significantly for the distance-based estimate when adjusted for running duration and other exercise (P = 3.2×10^−14^), but not for the time-based estimate when adjusted for distance and other exercise (P = 0.13). Moreover, the per METhr/d odds reduction was significantly greater for the distance- than the time-based estimate (P<10^−5^). Except for diabetes in females, the distance-based estimates were significantly related to reduced medication use when adjusted running duration. In addition, the odds reduction per METhr/d run was significantly greater for the distance- than the time-based estimate for female hypertensive medication use (P = 0.003), male and female high cholesterol medication use (P<10^−4^ and P = 0.02, respectively), and diabetes medication use in men (P = 0.001). In contrast, running duration was usually not significantly (male and female hypertension, male diabetes) or only weakly related (male and female high cholesterol) to the use of these medications when adjusted for distance.

### Walking

#### METhr/d walked vs. other exercise

Table 3 tests the exchangeability premise for walking versus other exercise among participants of the National Walkers' Health Study. When METhr/d for walking was estimated from distance, the decline in hypertension medication use per METhr/d was significantly greater for walking than “other exercise” in men (3.8-fold greater, P = 0.01) and women (4.9-fold greater, P = 1.9×10^−8^). The per METhr/d declines in diabetes medication use was also greater in men (8.8-fold greater, P = 0.002) and marginally greater in women (2.2-fold greater, P = 0.05). The results for high cholesterol medications was less consistent, with declining use being more strongly associated with walking than with other exercise in women (3.9-fold greater, P = 0.0003) but not men (2.4-fold greater, P = 0.46). However, as in the case of the runners, estimated energy expenditure from self-reported distance was more strongly related to declining medication use than its time-based estimate. When both walking and “other exercise” were calculated from self-reported exercise duration, the differences in medication use per METhr/d of exercise were eliminated or substantially reduced. The results were similar for walking compared to all “other exercise” and walking compared to “other moderate exercise”.

**Table 3:** Odds ratio (95% confidence interval) for hypertensive, high cholesterol, and diabetes medication use versus METhours per day of walking and other physical activities.

	Dependent variables:		
	Hypertension	High cholesterol	Diabetes
**Males**			
Distance-based estimate			
Walking_Distance_	0.93‡	0.97	0.87§
	(0.90, 0.97)	(0.94, 1.01)	(0.81, 0.94)
Other exercise	0.98*	0.99	0.99
	(0.96, 1.00)	(0.97, 1.01)	(0.96, 1.02)
*Walking_Distance_ vs. other exercise*	*P = 0.01*	*P = 0.46*	*P = 0.002*
Walking_Distance_	0.93§	0.97	0.87§
	(0.90, 0.97)	(0.94, 1.01)	(0.81, 0.94)
Vigorous exercise	0.97†	0.98	0.97
	(0.95, 0.99)	(0.96, 1.00)	(0.93, 1.01)
Other moderate exercise	1.00	1.00	1.01
	(0.97, 1.03)	(0.97, 1.03)	(0.96, 1.06)
Light exercise	1.02	0.99	1.00
	(0.92, 1.12)	(0.90, 1.10)	(0.87, 1.14)
*Walking_Distance_vs. other moderate*	*P = 0.006*	*P = 0.29*	*P = 0.0009*
Time-based estimate			
Walking_Time_.	0.98	1.01	0.93‡
	(0.95, 1.00)	(0.99, 1.04)	(0.88, 0.97)
Other exercise	0.98*	0.99	0.98
	(0.96, 1.00)	(0.97, 1.01)	(0.95, 1.01)
*Walking_Time_ vs. other exercise*	*P = 0.85*	*P = 0.10*	*P = 0.03*
Walking_Time_	0.98	1.01	0.92‡
	(0.95, 1.00)	(0.99, 1.04)	(0.88, 0.97)
Vigorous exercise	0.97†	0.98	0.96
	(0.95, 0.99)	(0.96, 1.01)	(0.92, 1.01)
Other moderate exercise	1.00	1.00	1.01
	(0.97, 1.03)	(0.97, 1.03)	(0.96, 1.06)
Light exercise	1.02	1.00	1.00
	(0.93, 1.13)	(0.91, 1.10)	(0.88, 1.15)
*Walking_Time_ vs. other moderate*	*P = 0.29*	*P = 0.53*	*P = 0.01*
**Females**			
Distance-based estimate			
Walking_Distance_	0.90§	0.94§	0.89§
	(0.88, 0.93)	(0.91, 0.96)	(0.85, 0.94)
Other exercise	0.98‡	0.98†	0.95†
	(0.97, 0.99)	(0.97, 1.00)	(0.92, 0.98)
*Walking_Distance_ vs. other exercise*	*P = 1.9×10^−8^*	*P = 0.0003*	*P = 0.05*
Walking_Distance_	0.90§	0.94§	0.89§
	(0.88, 0.92)	(0.91, 0.96)	(0.84, 0.94)
Vigorous exercise	0.98†	0.99*	0.96*
	(0.97, 0.99)	(0.97, 1.00)	(0.93, 1.00)
Other moderate exercise	0.98	0.99	0.94*
	(0.96, 1.01)	(0.97, 1.01)	(0.89, 1.00)
Light exercise	0.92	0.88†	0.81
	(0.84, 1.01)	(0.80, 0.97)	(0.62, 1.06)
*Walking_Distance_vs. other moderate*	*P = 1.1×10^−6^*	*P = 0.001*	*P = 0.16*
Time-based estimate			
Walking_Time._	0.96§	0.97§	0.93§
	(0.95, 0.98)	(0.95, 0.98)	(0.90, 0.96)
Other exercise	0.98‡	0.98†	0.95‡
	(0.96, 0.99)	(0.97, 0.99)	(0.92, 0.98)
*Walking_Time_ vs. other exercise*	*P = 0.10*	*P = 0.13*	*P = 0.41*
Walking_Time_	0.96§	0.97§	0.93§
	(0.95, 0.98)	(0.95, 0.98)	(0.90, 0.96)
Vigorous exercise	0.98†	0.98*	0.95†
	(0.96, 0.99)	(0.97, 1.00)	(0.92, 0.99)
Other moderate exercise	0.98	0.99	0.94*
	(0.96, 1.01)	(0.97, 1.01)	(0.89, 0.99)
Light exercise	0.92	0.88†	0.81
	(0.84, 1.01)	(0.79, 0.97)	(0.62, 1.05)
*Walking_Time_ vs. other moderate*	*P = 0.11*	*P = 0.14*	*P = 0.74*

Adjusted for age, education, current smoking status, and intakes of meat, fruit, and alcohol.

Significance of the regression coefficients coded.

* P<0.05;

† P<0.01;

‡ P<0.001;

§ P<0.0001;

¶ P<10^−15^,

in the model: Dependent variable = intercept+αWalking_Distance_+βOther exercise+covariates, or Dependent variable = intercept+αWalking_Time_+βOther exercise+covariates.

Abbreviations: BMI, body mass index; MET, metabolic equivalents of energy expenditure; Walking_Distance_, metabolic equivalent hr/d from walking as estimated from self-reported distance, Walking_Time_, metabolic equivalent hr/d from walking as estimated from self-reported duration.

#### Independent effects of time vs. distance estimates of METhr/d walked

Figures 1–3 further examine the differences between distance-based and time-based estimates of METhr/d walked and medication use by their direct comparison. They show that: 1) in all cases, distance walked was significantly related to declining medication use when adjusted for walking duration; 2) walking duration was only weakly related to medication use when adjusted for walking distance (exception diabetes medication use in women); 3) the reduction in male and female hypertensive medication use and male cholesterol medication use per METhr/d walked were significantly greater when estimated from distance than time.

### Running vs. Walking (analyses not displayed)

The combined analyses of runners and walkers showed that, with the exception of medication use for cholesterol lowering in men (runner vs. walker odds ratio: 0.93 vs. 0.98, P = 0.005 for difference) and to control hypertension in women (0.94 vs. 0.90, P = 0.02), the declines in the use of medications per METhr/d of exercise were generally not significantly different between running and walking. Specifically, when adjusted for covariates and other exercise, there were no significant differences for reductions in the use of medications to control male hypertension (runner vs. walker odds ratio: 0.92 vs. 0.93, P = 0.55 for difference), diabetes (males: 0.89 vs. 0.87, P = 0.65, females: 0.87 vs. 0.89, P = 0.70) and female high cholesterol (0.93 vs. 0.94, P = 0.46) per METhr/d run vs. per METhr/d walked.

## Discussion

Epidemiological studies rely primary on questionnaires to assess physical activity. These mostly record exercise duration and intensity [30]. The questionnaires vary in their ability to reproducibly quantify the activity dose and their validity as defined by their associations with health outcomes [30]. In general, vigorous physical activities have been shown to be more reproducibly reported than less-intense activities [31], [32]. Despite their limitations, cross-sectional populations surveys and prospective epidemiological follow-up studies have successfully used these questionnaires to identify a variety of health benefits for physical activity [2], [33]–[44], which has led to public health recommendations by government and scientific organizations [1]–[6]. However, there is little evidence that total energy expenditure as estimated in these questionnaires reflects the quality of exercise that confers its health benefits. In addition, large discrepancies between physical activity doses, as subjectively reported by participant questionnaires and objectively measured by accelerometers, demonstrate the need for improved self assessment [45], [46].

The current analyses address two fundamental questions concerning the epidemiology of exercise and its promotion: 1) whether exercise duration (time) is the right metric for quantifying walking and running, and 2) whether total energy expenditure is the appropriate metric for deducing the health effects of exercise. Existing public health recommendations and almost all published epidemiological studies estimate exercise dose as the cumulative total METminutes of activity as calculated from time and intensity. Nearly all physical activity questionnaires are based on assessing activity durations, and computing total expenditures based on the exchangeability premise [30]. In part, this approach is motivated by three factors: 1) the desire to create a simple algorithm that can be applied to all physical activities; 2) the desire to maximize flexibility such that participants can choose multiple activities to enhance compliance and researchers can combine activities to enhance statistical power; 3) the conviction that physical activity affects disease solely through energy expenditure without regard to intensity or mode. The third factor is an extension of a stated belief for physical activity and body weight [47], applied to other health outcomes.

This is not to deny that many studies show that greater total METminutes of physical activity predict lower risk for cardiovascular disease and other health outcomes [2]. Indeed, the analyses of Tables 2 and 3 show that except for hypertension and high cholesterol in the relatively small sample of male walkers, both Walking_time_ and Running_time_ were consistently associated with lower medication use for treating hypertension, high cholesterol and diabetes, in agreement with prospective data [9]. However, our analyses suggest that the time-based estimates of METhr/d of running and walking may underestimate the associations with disease status by 50% or more, and that a simple alternatives based on running and walking distances are better. Time-based estimation and prescription of physical activity may be too subjective, which may explain in part the substantial discrepancy between sample estimates of physical activity when assessed by questionnaires and when measured by accelerometers [48], [49].

An activity session often consists of less-active periods before and after the actual activity (preparation, warm-up, cool down, etc) and intermittent sedentary periods during the activity (e.g., transitioning between activities during a workout, breaks, etc). Estimates of running or walking that are distance-based should be relatively unaffected by such sedentary periods, whereas time-based estimates of running, walking, and other exercise could be substantially overestimated by the failure to exclude these inactive periods. For example, immediately following a one-hour surreptitious observational period, Klesges et al found that subjects significantly underestimated the proportion of time that they were sedentary, and significantly overestimated that in which they were aerobically active [50]. Observed and self-reported physical activity were only moderately correlated (r = 0.62), with 69% of subjects overestimating, 18% reporting accurately, and 13% underestimating the time spent being aerobically active. Aerobic activity was over-reported by greater than 300% in that study. These large discrepancies occurred even though the observation period was only an hour long and the recall was solicited immediately following the activity.

The analyses of Tables 2 and 3 suggests that running and walking may be more appropriately described by distance than time, and that for these activities, distance is a better metric than duration. Because walking is the most common exercise in the United States [51], and running among the more common vigorous exercises [51], a simple improvement in their prescriptions could substantially improve efforts to manage hypertension, high cholesterol, and diabetes, e.g., by simply reformulating the guidelines to specify running and walking targets by distance (e.g., 1.75 miles) rather than time (30 minutes). The benefits should be balanced against the risk of injuries associated with running, although in general runners then to be successful in sustaining their commitment to running or to other regular exercise over the long term [52].

These results are germane to understanding the success in associating health outcomes with exercise dose in the National Runners' and Walkers' Health Studies. These two studies are unique among all large epidemiological cohorts in targeting specific physical activities in their recruitment. Their analyses have shown that longer reported distances run and walked per week were associated with significantly lower body weight and circumferences of the waist, hip and chest [10]–[12], and significantly lower use of medications for controlling hypertension, high cholesterol, and diabetes [8], [13]. Prospectively, longer distances run per week were associated with significantly lower risks for weight gain [14], [15] and incident diabetes [9], hypertension [9], hypercholesterolemia [9], diverticular disease [16], gallbladder disease [17], gout [18], coronary heart disease [19], stroke [20], age-related macular degeneration [21], glaucoma [22], and cataracts [23]. The success in identifying these associations is likely due in part to the statistical power afforded by the study's large sample size and broad range of activity. However, it also appears that reported METhr/d of running is more strongly associated with health benefits than equivalent energy expenditure from other reported exercise. Moreover, the MET values published in the compendium of physical activities [7] suggest that energy expenditure by running is a simple function of distance irrespective of intensity or duration [24]. Thus, the success in identifying disease associations in the National Runners' Health Study could also be due to the more accurate self-assessment of activity dose when reporting usual distance run than when reporting usual duration per week and pace.

A key premise of all the recommendations cited above is the “exchangeability premise”, i.e., that various physical activities can be combined to meet recommendations based on their calculated energy expenditures [1]–[6]. The analyses suggest that the exchangeability premise largely held when comparing running with walking, which may be the best test of the equivalence of moderate vs. vigorous intensity exercise. There were substantial differences in the odds for hypertension, hypercholesterolemia, and diabetes between runners and walkers, however except for men's use of cholesterol lowering medications (P = 0.005), and women's use of hypertension medication (P = 0.02), there were no significant difference in the decreased odds per METhr/d run versus walked. Table 3 showed that there were also only modest differences between walking and other moderate intensity exercise in their associations with medication use. Thus the exchangeability premise would appear to generally hold for hypertension, hypercholesterolemia, and diabetes.

Tables 2 showed that the decline in medication use with increasing METhr/d of exercise was greater for running than for other exercise, including other vigorous exercise. This is consistent with other evidence showing that reductions in men's cardiovascular disease risk and other health outcomes are greater for vigorous than less-intense exercise [33]–[35]. This result is not necessarily a contradiction of the lack of significant difference between running and walking in that walking represented a third of the other exercise reported by runners. Running and walking generally affect the same muscle groups and share other characteristics that may distinguish them from other exercises. The health benefits per METhr/d may be greater for running than other non-walking exercise. It is also possible that self-assessment is more accurate for running than for other exercise, including other vigorous exercise. In either case, a physical activity target defined in terms of running could yield greater health benefits than a target defined in terms of other exercise.

### Caveats

The runners and walkers used in these analyses were recruited as potential participants for a clinical trial and therefore may not be representative of the general population. The low smoking rates and relatively high educational attainment both suggest a more health conscious and educated sample than the general population. Others have also shown that habitual runners exhibit healthier behavioral risk profiles than nonrunners [53]. In addition, the goal of re-surveying the sample was not to maximize response rates but rather to obtain as large a sample as possible. Therefore, it remains to be determined whether the current findings apply to other populations. There is also an inherent limitation of cross-sectional analyses in that it is uncertain whether physical activity proceeded medication use or whether the converse occurred. The current analyses examines, within individuals, the differences of two different metrics for quantifying the amount of running and walking performed, and their relationship to medication use. Presumably any tendency for medication use to affect physical activity would affect both metrics, as well as other exercise. Moreover, prospective follow-up of the initial cohort showed that longer running distances did precede incident use of these medications [9]. Recognized biases in self-reported physical activity include over-reporting (social desirability bias) and inherent difficulties in assessing intensity [54], [55]. However, the same intensity factors were used for both the time-based and distance-based calculations of METhr/d run or walked, so this should not affect their comparison. It is unlikely that the lower use of medications to control hypertension, hypercholesterolemia, and diabetes with running and walking distance is due to less screening for these conditions in more physically active men and women. The Health Professional Study reported that their more vigorously active participants had more routine medical check-ups than less active men [56], and activity level was not reported to affect the frequency of routine medical check-ups in the Nurses' Health Study [57].

In addition, it is acknowledged that men and women who regularly run or walk for exercise may quantify their activity differently than those who do not. In particular, habitual walkers and runners may recall their preferred exercise (e.g., walking and running) better than recall of physical activity in general among non-habitual exercisers, which may also limit generalization. Walking and other training activities for keeping in shape may be better reported because they are regularly performed with less variation than daily activities not specifically part of a training regimen. “Walking for exercise” has also been shown by others to be more predictive of falling within an above-average fitness category than more-inclusive walking assessment [58]. For this reason our results, based on walking for exercise in self-identified walkers, may not necessarily apply to less-structured walking incorporated into daily living. In addition, the ability to quantify walking may be less in persons who do not regularly walk for exercise [59]. For example, the reported correlations between physical activity records and individual activities in the Minnesota leisure time physical activity questionnaire were substantially weaker for walking (r = 0.25) than running (r = 0.82) [59]. Moreover, the average mean minutes per day walked was about 8-fold greater from the physical activity record than the Minnesota Leisure time physical activity questionnaire.

Non-habitual exercisers may also overestimate their time-based exercise dose by overestimating the intensity of their activity. Less-fit subjects, in particular, appear to overestimate the intensity of the activities they perform, even among moderately intense physical activities [60]. Finally, in interpreting differences between running and walking, and between these exercises and other physical activities, is that intra-class correlations for agreement tend to be stronger for walking and vigorous exercise than for other non-vigorous exercise. Other limitations affecting the more traditional time and intensity-based estimation of physical activity dose have been discussed [55], [61]. Thus it is not known whether the same results would have been obtained in a sample unaccustomed to regular walking and running. There is a need to replicate these results in other populations.

### Conclusions

The success of the National Runners' Health Study to identify associations between running and health outcomes at greater exercise doses than previously studied probably relates in part to the superiority of the distance-based metric for quantifying running. The analyses suggest that the calculations of walking and running energy expenditures from reported distance are more strongly related to medication use than their traditional time-based calculations, and that the estimated effect per METhr/d of exercise may be greater in some cases for running than other exercise. The results suggest that distance run or walked may provide a better research metric for epidemiologic research, and better public health targets, than running and walking duration.
